# Comparison of two methods of extracting bull epididymal spermatozoa

**DOI:** 10.1016/j.vas.2024.100407

**Published:** 2024-11-06

**Authors:** Ziyad Al-Kass, Sanna Eriksson, Jaana Peippo, Theodoros Ntallaris, Jane M. Morrell

**Affiliations:** a Department of Clinical Sciences, Swedish University of Agricultural Sciences, Box 7054, SE-75007 Uppsala, Sweden; b Department of Surgery and Theriogenology, College of Veterinary Medicine, university of Mosul, Mosul, Iraq; c Nordic Genetic Resource Center (NordGen), c/o NMBU – Biovit Box 5003, 1432 Ås, Norway

**Keywords:** Sperm freezing, Cooled semen, Sperm recovery, Bovine

## Abstract

Extraction of epididymal spermatozoa may be necessary to avoid losing valuable genetic material, for example, from individuals of rare breeds or endangered species, but the resulting sperm samples may be of poor quality. Two methods of extracting bull epididymal spermatozoa from slaughterhouse material were compared. The bulls were 16–23 months of age. Spermatozoa were extracted by making an incision one cm in length in the tail of the epididymis to allow the spermatozoa to flow out (method A), or by flushing the tail of epididymis (method B). The two methods were used for each bull, alternating between right and left epididymis, i.e. if method A was used for the left epididymis in Bull 1, it was used for the right epididymis in bull 2, etc. Sperm concentration in the extracted samples was adjusted to 69 × 10^6^/mL in Andromed; the sperm sample was packed in 0.25 mL straws. After cooling for two h at 5 °C, the straws were placed 4 cm above liquid nitrogen for 20 min before transferring them to liquid nitrogen. Sperm motility, viability, reactive oxygen species, membrane integrity and DNA fragmentation were analysed in the fresh samples and again after thawing. The results for all parameters in fresh semen were not different between methods. Although sperm quality was lower in thawed samples than in fresh samples, there was no difference in sperm quality between the two extraction methods in the thawed samples. In conclusion, both methods are useful for the extraction of bull epididymal spermatozoa.

## Highlights

• Cutting and flushing methods of extracting bull epididymal spermatozoa were compared.
• Proportion of dying spermatozoa was greater for the cutting method than for flushing.
• Otherwise, sperm quality in fresh samples was similar between extraction methods.
• Sperm quality in thawed samples was similar between cutting and flushing methods.
• Both methods could be useful for the extraction of epididymal bull spermatozoa.

## Graphical abstract

**Figure:**
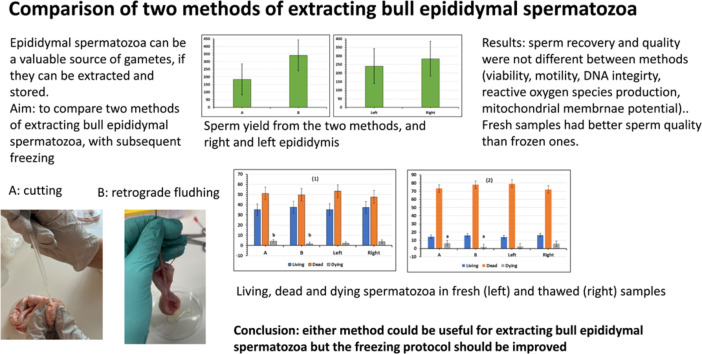

## 1 Introduction

Spermatozoa in the testes are immature and cannot fertilize an oocyte, but they undergo maturation as they pass through the epididymis (Jones & Lopez, 2004). Anatomically, the epididymis consist of four regions, the initial segment, caput, corpus, and cauda, each of which has a unique function and characteristics (James et al., 2020). When spermatozoa enter the epididymis, they are incapable of motility and cannot capacitate. They mature during epididymal passage, for example by releasing and absorbing ions, antioxidants, and fluids (Trigg et al., 2019). By the time they arrive in the cauda epididymis, they are capable of becoming motile when activated and are sufficiently mature to be able to undergo capacitation in the right conditions. Most of the mature epididymal spermatozoa, 50 % to 80 %, are stored in the cauda epididymis (Cornwall, 2009). Spermatozoa can be recovered successfully in seasonal breeders but also from non-seasonal breeders such as stallions (Talluri et al., 2023), dogs (Mogheiseh et al., 2022), bulls (Kang et al., 2018), bucks (Abu et al., 2016), cats (Prochowska et al., 2016), and deer (Koziol & Koziorowski, 2015).

Secretions from male accessory genital glands are mixed with spermatozoa from the epididymis on ejaculation. These secretions include proteins, lipids, ions, organic and non-organic material, which are important for transportation and sperm survival within the female reproduction tract (Juyena & Stelletta, 2012). Thus, there are considerable differences between ejaculated and epididymal spermatozoa. However, the collection of epididymal spermatozoa from animals after death can be an important source of gametes, especially from rare breeds and endangered species. It could also be useful in the event of the death of a valuable production animal, representing the last chance to obtain his gametes (Martins et al., 2007). Therefore, even in species such as cattle, where semen is routinely collected using an artificial vagina, a method of recovering epididymal spermatozoa could be a valuable method for rescuing gametes.

Epididymal spermatozoa can be used for insemination fresh or frozen (Gilmore et al., 1998). In a study by Nazari et al. (2020), thawed epididymal sperm were able to fertilizing oocytes, although they might have lower motility, velocity, linearity, and straightness than ejaculated sperm (Goovaerts, 2006).

Several collection methods were reported for epididymal spermatozoa, involving different methods of releasing the spermatozoa. In the cutting method, several incisions are made in the caudal epididymis to allow the spermatozoa to exit (Santiago-Moreno et al., 2009). In the float-up method (Turri et al., 2012), spermatozoa seep out from the cut surface into medium. In the retrograde flushing method (Martinez-Pastor et al., 2006), medium is flushed through the vas deferens into the most caudal part of the cauda epididymis. Spermatozoa can be aspirated directly from the epididymis using a microsurgical technique (Bernie et al., 2013). A squeezing method can be used where pressure is applied to the tissue to force the spermatozoa out (Damm & Cooper, 2010). Generally, methods that involve manipulation of the tissue can cause contamination with blood and other cells, which are detrimental to sperm survival (Muñoz-Fuentes et al., 2014).

Several studies showed the benefit of using epididymal spermatozoa for in vitro embryo production (Bertol et al., 2016; Krishnakumar et al., 2011). However, the quality and viability of epididymal spermatozoa vary according to the age of the animal (Turri et al., 2012), the size of the epididymis (Palasz et al., 1994), and the method of collection (Talluri et al., 2023). Sperm quality was reported to be better after the flushing method than after the float-up technique (Turri et al., 2012).

The objective of this study was to collect and freeze bull epididymal spermatozoa using two of the methods just described, namely the cutting method and the retrograde flushing method. The number of spermatozoa recovered by each method and the quality of the resulting samples were assessed, both immediately after harvesting and after freezing and thawing.

## 2 Material and methods

### 2.1 Experimental design

**Fig. 1:**
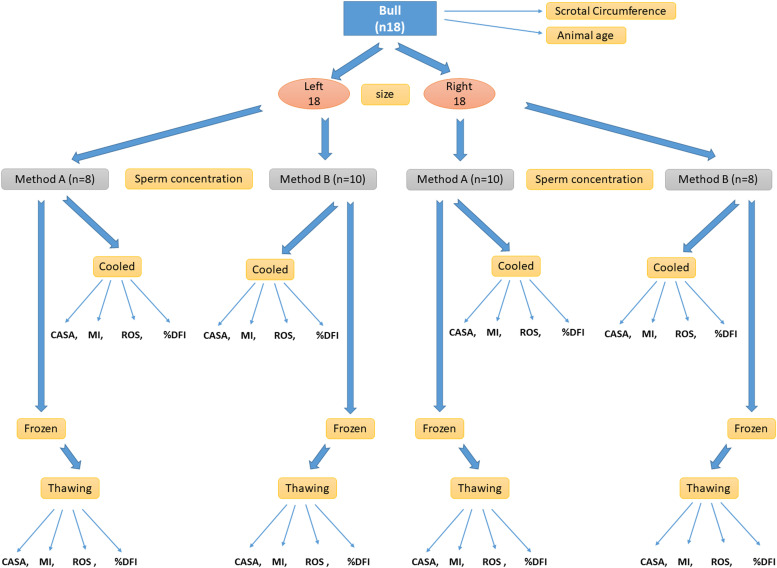
Experimental design, comparing two methods of extracting bovine epididymal spermatozoa from slaughterhouse material. The extraction methods were used alternately between right and left epididymis for 18 bulls. Abbreviations: MI, Membrane Integrity; %DFI, DNA fragmentation index; ROS, Reactive Oxygen Species; and CASA, Computer-Assisted Sperm Analysis.

The experimental design is shown in Fig. 1.

### 2.2 Animals

The scrotum from each of 18 bulls, 16–23 months of age, were obtained from a local slaughterhouse (Lövsta slaughterhouse, Uppsala, Sweden). The breeds were Angus x Hereford (2), Angus (3), Hereford (12), and one not reported. The organs were transported to the laboratory in an insulated box at ambient temperature (approximately 18 °C) not more than two hours after slaughter. Scrotal circumference and testis size were measured. The epididymis was carefully separated from the surrounding tissues. Measurements for the right and left testes from each pair were recorded, size was calculated, and any lesions or haemorrhage noted. One epididymis from each pair was allocated to cutting, the other to flushing, alternating between bulls. All procedures were performed in compliance with relevant laws and institutional guidelines; use of slaughterhouse material does not require ethical approval in Sweden, as confirmed by the appropriate institutional committee.

### 2.3 Sperm collection

Extraction of sperm was achieved by two methods. In the first method (A), an incision one cm in length was made in the tail of the epididymis to allow the sperm to seep out (Cunha et al., 2016). The extruded sperm were collected in a plastic pipette and were transferred to one mL of extender (Andromed; Minitube International, Tiefenbach, Germany).

**Fig. 2:**
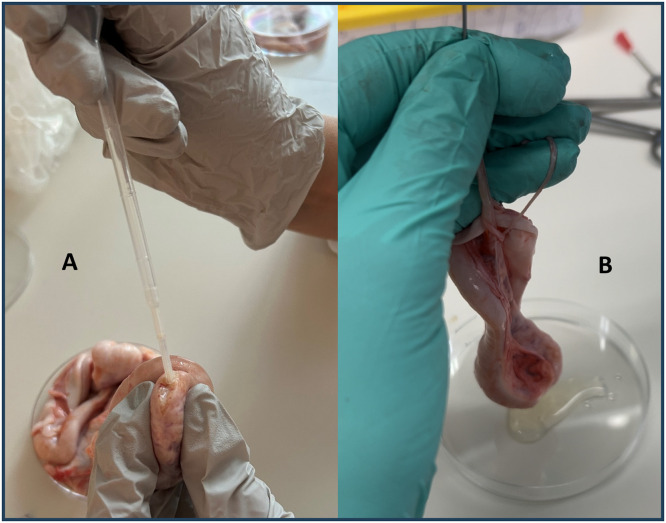
Methods for sperm extraction from bovine epididymis: A incision in tail of epididymis, and B flushing the tail of epididymis.

The second method (B) was performed by inserting a blunt 18-gauge needle into the spermatic cord, making an incision in the tail of epididymis and flushing 5 mL of Andromed extender in a retrograde manner through the vas deferens and part of the cauda epididymis (Fig. 2).

### 2.4 Sperm concentration

Sperm concentration was measured using the Nucleocounter-SP 100 (Chemometec, Allerød, Denmark), following the manufacturer´s instructions.

### 2.5 Sperm cryopreservation

Sperm concentration was adjusted to 69 × 10^6^/mL with Andromed, except for three samples where the initial sperm concentration was already lower than this value. The samples were equilibrated for 2 h at 5 °C before manually filling 0.25 mL straws (CRYO-VET France) in a cold bench at 5 °C. The straws were placed on a rack 4 cm above the surface of liquid nitrogen for 20 min in a 40 × 30 cm box before transferring them to liquid nitrogen until required for analysis.

### 2.6 Thawing

Straws were thawed in a water bath at 37 °C for 12 s.

### 2.7 Sperm evaluation

Samples were evaluated immediately before cryopreservation and again after thawing:

#### 2.7.1 Computer-assisted sperm analysis (CASA)

Sperm motility was analysed using AndroVision® (Minitüb Abfüll-und labortechnik GmbH and Co.KG, Tiefenbach, Germany), after incubation at 37 °C for 5 min. At least four fields (approximately 1000 spermatozoa in total) were analysed using AndroVision software for the following kinematics: Beat cross frequency (BCF, Hz), linearity (LIN), lateral head displacement (ALH, µm), wobble (WOB), velocity of the average path (VAP, µm/s), straight-line velocity (VSL, µm/s), straightness (STR), curvilinear velocity (VCL, µm/s), in addition to progressive motility (PM, %) and total motility (TM, %). The settings for bull spermatozoa were as follows: slow motility VCL < 120.00; local motility VCL < 40.00; immotile sperm HAC < 0.087; circle motility radius > 10.00; VSL < 10.00; and radius < 60.00 and rotation > 0.70.

#### 2.7.2 Flow cytometry, lasers and filters

All samples were analysed using a FACSVerse™ flow cytometer (BD Biosciences, Becton Dickinson and Company, San Jose, CA, USA). For fluorescent stains, a violet laser at 405 nm and a blue laser at 488 nm were used. The bandpass filters for detecting fluorescence were green (527/32 nm), orange (586/42 nm), red (700/32 nm), and blue (528/45 nm).

##### 2.7.2.1 Membrane integrity (MI)

Membrane integrity was analysed by flow cytometry after staining with 12 μM propidium iodide (PI) and 0.02 μM SYBR14 (Live-Dead Sperm Viability Kit L-7011; Invitrogen, Eugene, OR, USA). Sperm concentration was adjusted to 2 × 10^6^ spermatozoa/mL, before 300 μL was stained with SYBR14 (0.5 μL) and 3 μL PI, and incubated at 38 °C for 10 min Cojkic et al. (2023). The sperm were classified as having an intact membrane/living (stained with SYBR14 only) or damaged membranes spermatozoa/ Dead and Dying (stained with PI, SYBR14 negative or positive).

##### 2.7.2.2 Reactive oxygen species (ROS)

Sperm concentration was adjusted to 2 × 10^6^ sperm/ml; 300 μL were stained using 3 μL (40 mM) Hoechst 33,258 (HO) (Sigma, Stockholm, Sweden), 3 μL (40 mM) Hydroethidine (HE) (Invitrogen, Thermo Fisher Scientific, Eugene, OR, USA), and 3 μL (2 mM) of 20, 70 -dichlorodihydrofluorescein diacetate (DCFDA) (Invitrogen, Thermo Fisher Scientific, Eugene, OR, USA). Samples were mixed gently, incubated at 38 °C for 30 min, and analysed by flow cytometry (FC), as described above.

The proportions of live superoxide positive (live SO+) and negative (live SO−), live hydrogen peroxide positive (live H_2_O_2_+), and negative (live H_2_O_2_−), dead superoxide positive (dead SO+), and dead hydrogen peroxide positive (Dead H_2_O_2_+) and negative (Dead H_2_O_2_−) were calculated after gating out debris.

##### 2.7.2.3 Sperm chromatin structure assay (SCSA)

Samples were prepared for SCSA by mixing 50 μL sperm with 50 μL buffer solution composed of 0.01 M tris HCL, 0.15 M sodium chloride and 1 mM Ethylene diaminetetraacetic acid (TNE buffer) at pH 7.4. The samples were frozen in liquid nitrogen and stored at – 80 °C.

Samples were slowly thawed on ice; aliquots (10 μL) were mixed with 90 μL TNE buffer, and 200 μL acid-detergent solution. After 30 s, 600 µL acridine orange (AO) was added (Evenson and Jost, 2001). The samples were analysed by flow cytometry

### 2.8 Statistical analysis

All analysis were run using SAS software (version 9.4; SAS Institute, Cary, NC). Data distribution was checked using the Kolmogorov-Smirnov test. Average values (mean, standard deviation, min, max, and boxplots) were calculated with the MEANS and SGPLOT functions in SAS. Sperm data from bulls were analysed with the PROC MIXED function. Not normally distributed variables were log-transformed (Age, concentration, TM, PM, VCL, VSL, VAP, and other sperm-related measures). However, they are shown in the manuscript as their original untransformed versions for easier understanding.

The least squares means (LSM ± SEM) from models were compared using Scheffé’s method for adjusting multiple comparisons after ANOVA. Fixed effects included Method (2 levels), Side (left or right), Breed (3 types), and their interactions. The random effect was Animal.

To find correlations among sperm traits, the CORR function was used. The 22 traits were grouped by a Factor analysis, using Varimax for rotation. This analysis identified 2 major components, grouping traits based on the highest positive value in the rotated component matrix.

A 5 % alpha level was used, and p-values were compared to this threshold. Any differences where 0.05 < *p* ≤ 0.10 were noted as trends.

## 3 Results

### 3.1 Sperm count

**Fig. 3:**
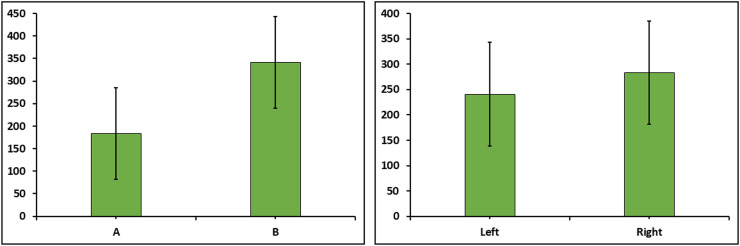
Least Squares Means ± SE for sperm count (million spermatozoa/mL) after extracting spermatozoa from bovine epididymis, according to method, the incision method (A) or the flushing method (B), or according to left or right epididymis (*n* = 14).

The data from 4 animals were excluded due to adhesion or haemorrhage in the tissue. The total number of spermatozoa obtained was not different between the two methods or between left and right sides (Fig. 3).

### 3.2 Sperm motility

Sperm kinematics are shown in (Table 1: Fresh samples, and Table 2: Post thaw samples), according to method of collection and source, i.e. Right or Left epididymis. There were no significant differences in TM between method A and Method B for Fresh or for frozen sperm samples, or for Left and Right side. Similarly, there were no differences in PM between method A and B or between Left and Right. Furthermore, there were no differences in the other sperm kinematics except BCF for frozen spermatozoa, which was higher for method B than method A (*p* < 0.05).

**Table 1:** Sperm kinematics for fresh bull epididymal samples collected from left and right epididymides using two different methods, incision in tail of epididymis (A), and flushing the tail of epididymis (B). data shown are Least Squares Means ± Standard Error (*n* = 18).

**Kinematics**	**Method A**	**Method B**	**Left**	**Right**
TM (%)	40.7 ± 5.7	48.2 ± 5.7	42.4 ± 5.7	46.5 ± 5.7
PM (%)	39.3 ± 5.7	47.1 ± 5.7	41.3 ± 5.7	45.2 ± 5.7
VCL (µm/s)	91.2 ± 15.7	118.6 ± 15.7	99. 9 ± 15.7	109.9 ± 15.7
VSL (µm/s)	33.1 ± 6.6	44.5 ± 6.6	37.0 ± 6.6	40.6 ± 6.6
VAP (µm/s)	41.9 ± 7.9	54.7 ± 7.9	45.8 ± 7.9	50.7 ± 7.9
ALH (µm)	1.0 ± 0.2	1.3 ± 0.2	1.1 ± 0.2	1.2 ± 0.2
BCF (Hz)	6.9 ± 0.6	7.6 ± 0.6	7.0 ± 0.6	7.6 ± 0.6
WOB	0.4 ± 0.02	0.4 ± 0.02	0.4 ± 0.02	0.4 ± 0.02
LIN	0.3 ± 0.02	0.3 ± 0.02	0.3 ± 0.02	0.3 ± 0.02
STR	0.8 ± 0.02	0.8 ± 0.02	0.8 ± 0.02	0.8 ± 0.02

Note: TM, total motility; PM, progressive motility; VCL, curvilinear velocity; LIN, linearity; VSL, straight-line velocity; VAP, velocity of the average path; STR, straightness; ALH, lateral head displacement; BCF, beat cross frequency; WOB, wobble.

**Table 2:** Post-thaw sperm kinematics for bull epididymal samples collected from left and right epididymides using two different methods, incision in tail of epididymis (A), and flushing the tail of epididymis (B). data shown are Least Squares Means ± Standard Error (*n* = 18).

**Kinematics**	**Method A**	**Method B**	**Left**	**Right**
TM (%)	20±2.6	24±2.6	19.7 ± 2.6	24.1 ± 2.6
PM (%)	18.4 ± 2.5	22.6 ± 2.5	18.6 ± 2.5	22.4 ± 2.5
VCL (µm/s)	41.3 ± 4.3	49.7 ± 4.3	41.7 ± 4.3	49.4 ± 4.3
VSL (µm/s)	12.7 ± 1.7	15.1 ± 1.7	12.7 ± 1.7	15.1 ± 1.7
VAP (µm/s)	17.3 ± 2.1	20.3 ± 2.1	17.3 ± 2.1	20.4 ± 2.1
ALH (µm)	0.5 ± 0.05	0.6 ± 0.05	0.5 ± 0.05	0.6 ± 0.05
BCF (Hz)	3.8 ± 0.5^a^	5.0 ± 0.5^a^	4.2 ± 0.5	4.6 ± 0.5
WOB	0.4 ± 0.02	0.4 ± 0.02	0.4 ± 0.02	0.4 ± 0.02
LIN	0.3 ± 0.02	0.3 ± 0.02	0.3 ± 0.02	0.3 ± 0.02
STR	0.7 ± 0.03	0.7 ± 0.03	0.7 ± 0.03	0.7 ± 0.03

Note: TM, total motility; PM, progressive motility; VCL, curvilinear velocity; LIN, linearity; VSL, straight line velocity; VAP, velocity of the average path; STR, straightness; ALH, lateral head displacement; BCF, beat cross frequency; WOB, wobble. Similar superscript letters within a row refer to a significant difference, *p* < 0.05.

### 3.3 Viability

**Fig. 4:**
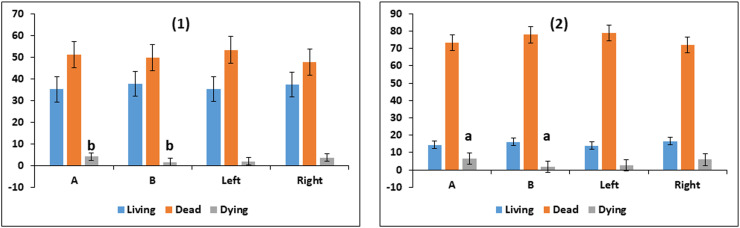
Living, dead, and dying bull epididymal sperm samples (Least Squares Means ± SE) extracted by the cutting method (A) or flushing method (B); and left or right sides. fresh samples (1) and frozen samples (2) (*n* = 18). (Similar superscript letters refer to significant differences: ^a^*p* < 0.05, ^b^*p* < 0.02.

The proportions of living and dead spermatozoa were not different between the two methods for either fresh or frozen samples. Similarly, living and dead spermatozoa were not different between Left and Right (Fig. 4). However, the proportion of dying spermatozoa was different between the two extraction methods, being 4.1 ± 1.7 % and 1.5 ± 1.7 %, for cutting and retrograde flushing, respectively in fresh samples (*p* < 0.02); and 6.6 ± 3.3 and 1.9 ± 3.3, respectively, in frozen samples (*p* < 0.05). There were no differences between Left and Right.

### 3.4 Reactive oxygen species

The ROS status (Table 3: Fresh samples, and Table 4: post-thaw samples) did not differ between methods or between sides for fresh and frozen spermatozoa.

**Table 3:** Reactive oxygen species in fresh bull epididymal spermatozoa for samples collected from left and right epididymides by two methods: incision in tail of epididymis (A) or retrograde flushing of the tail of epididymis (B) (Least Squares Means ± Standard Error; *n* = 18).

**Parameters**	**Method A**	**Method B**	**Left**	**Right**
Live H_2_O_2_ +	0.5 ± 0.1	0.3 ± 0.1	0.3 ± 0.1	0.3 ± 0.1
Live H_2_O_2_-	61.1 ± 4.8	65.1 ± 4.8	60.5 ± 4.8	65.7 ± 4.8
Dead H_2_O_2_+	1.0 ± 0.5	1.5 ± 0.5	1.1 ± 0.5	50.7 ± 0.5
Dead H_2_O_2_-	37.8 ± 4.9	33.9 ± 4.9	38.4 ± 4.9	33.3 ± 4.9
Live SO +	13.2 ± 2.7	14.3 ± 2.7	13.5 ± 2.7	14±2.7
Live SO -	47.8 ± 6.2	51.7 ± 6.2	46.9 ± 6.2	52.5 ± 6.2
Dead SO +	38.5 ± 4.8	34.8 ± 4.8	39.4 ± 4.8	33.9 ± 4.8

Notes: H_2_O_2_ = hydrogen peroxide, SO = superoxide.

**Table 4:** Reactive oxygen species in thawed bull epididymal spermatozoa collected from left and right epididymides by two methods: incision in tail of epididymis (A) or retrograde flushing of the tail of epididymis (B) from left and right epididymis (Least Squares Means ± Standard Error; *n* = 18).

**Parameters**	**Method A**	**Method B**	**Left**	**Right**
Live H_2_O_2_ +	0.08 ± 0.02	0.06 ± 0.02	0.07 ± 0.02	0.08 ± 0.02
Live H_2_O_2_ -	42.3 ± 4.4	47.2 ± 4.4	41.0 ± 4.4	48.4 ± 4.4
Dead H_2_O_2_ +	0.2 ± 1.0	1.2 ± 1.0	0.3 ± 1.0	1.1 ± 1.0
Dead H_2_O_2_-	57.4 ± 4.4	52.6 ± 4.4	58.7 ± 4.4	51.4 ± 4.4
Live SO +	20.4 ± 3.8	24.6 ± 3.8	21.3 ± 3.8	23.7 ± 3.8
Live SO -	21 ± 3.4	23.8 ± 3.4	18.8 ± 3.4	25.9 ± 3.4
Dead SO +	57.3 ± 4.5	52.9 ± 4.5	59.2 ± 4.5	51.0 ± 4.5

Notes: H_2_O_2_ = hydrogen peroxide, SO = superoxide.

### 3.5 The DNA fragmentation index

**Fig. 5:**
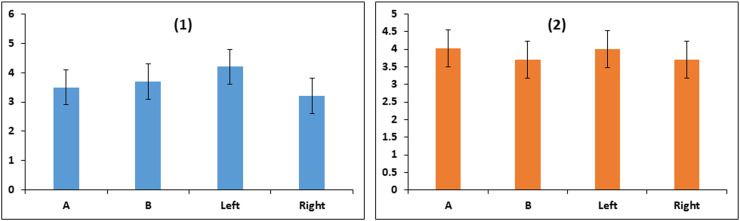
DNA fragmentation index (%) in bull epididymal sperm samples according to extraction method (incision, A, or flushing, B) and side (left or right), fresh samples (1) and frozen samples (2) results are shown as Least Squares Means ± SE (*n* = 18).

The %DFI was not different between extraction methods or between Left and right sides (Fig. 5, (1) Fresh, and (2) thawed samples).

### 3.6 Correlations

There were significant positive correlations for fresh spermatozoa between TM and membrane integrity (*p* < 0.0001), and between TM and most ROS sub-populations except for dead H_2_O_2_ +, which was not significant (Table 5). There was a trend towards significance for a negative correlation between TM and %DFI. However, for thawed spermatozoa (Table 6), there was a significant correlation between TM and membrane integrity (*p* < 0.007), %DFI (*p* < 0.05) live H_2_O_2_ -, dead H_2_O_2_ + and dead SO + (*p* < 0.0001), as well as live SO- (*p* < 0.002).

**Table 5:** Correlations between total motility, membrane integrity, DNA fragmentation and reactive oxygen species production in fresh bull epididymal sperm samples (*n* = 18).

	**Variable (%)**	**TM (%)**	**MI (%)**	**%DFI**
**1**	**Live H_2_O_2_ –**	R^2^ = 0.91, *p* < 0.0001	R^2^ = 0.85, *p* < 0.0001	R^2^ = -0.26, *p* < 0.028
**2**	**Live H_2_O_2_ +**	R^2^ = 0.63, *p* < 0.0001	R^2^ = 0.62, *p* < 0.0001	R^2^ = 0.25, *p* < 0.86
**3**	**Dead H_2_O_2_ -**	R^2^ = -0.92, *p* < 0.0001	R^2^ = -0.85, *p* < 0.0001	R^2^ = -0.09, *p* < 0.035
**4**	**Dead H_2_O_2_ +**	R^2^ = 0.15, *p* < 0.22	R^2^ = 0.17, *p* < 0.165	R^2^ = -0.125, *p* < 0.43
**5**	**Live SO -**	R^2^ = 0.93, *p* < 0.0001	R^2^ = 0.88, *p* < 0.0001	R^2^ = -0.26, *p* < 0.29
**6**	**Live SO +**	R^2^ = -0.62, *p* < 0.0001	R^2^ = -0.55, *p* < 0.0001	R^2^ = -0.26, *p* < 0.08
**7**	**Dead SO +**	R^2^ = -0.91, *p* < 0.0001	R^2^ = -0.83, *p* < 0.0001	R^2^ = -0.26, *p* < 0.03
**8**	**MI**	R^2^ = 0.91, *p* < 0.0001		R^2^ = -0.26, *p* < 0.028
**9**	**%DFI**	R^2^ = -0.23, *p* < 0.05	R^2^ = -0.26, *p* < 0.028	

Note: TM = Total Motility; MI = membrane integrity; H_2_O_2_ = hydrogen peroxide; SO^.^ = superoxide; %DFI = DNA fragmentation index.

**Table 6:** Correlations between total motility, membrane integrity, DNA fragmentation and reactive oxygen species production in thawed bull epididymal sperm samples (*n* = 18).

	**Variable (%)**	**TM (%)**	**MI(%)**	**%DFI**
**1**	**Live H_2_O_2_ -**	R^2^ = 0.70, *p* < 0.0001	R^2^ = 0.85, *p* < 0.0001	R^2^ = - 0.26, *p* < 0.028
**2**	**Live H_2_O_2_ +**	R^2^ = 0.09, *p* < 0.57	R^2^ = 0.62, *p* < 0.0001	R^2^ = 0.25, *p* < 0.86
**3**	**Dead H_2_O_2_ -**	R^2^ = - 0.92, *p* < 0.0001	R^2^ = -0.85, *p* < 0.0001	R^2^ = - 0.09, *p* < 0.035
**4**	**Dead H_2_O_2_ +**	R^2^ = 0.15, *p* < 0.22	R^2^ = 0.17, *p* < 0.165	R^2^ = - 0.125, *p* < 0.43
**5**	**Live SO -**	R^2^ = 0.93, *p* < 0.0001	R^2^ = 0.88, *p* < 0.0001	R^2^ = - 0.26, *p* < 0.29
**6**	**Live SO +**	R^2^ = -0.62, *p* < 0.0001	R^2^ = -0.55, *p* < 0.0001	R^2^ = - 0.26, *p* < 0.08
**7**	**Dead SO +**	R^2^ = 0.91, *p* < 0.0001	R^2^ = -0.83, *p* < 0.0001	R^2^ = - 0.26, *p* < 0.03
**8**	**MI**	R^2^ = 0.91, *p* < 0.0001		R^2^ = - 0.26, *p* < 0.028
**9**	**%DFI**	R^2^ = -0.23, *p* < 0.05	R^2^ = - 0.26, *p* < 0.028	

Note: TM = Total Motility; MI = membrane integrity; H_2_O_2_ = hydrogen peroxide; SO^.^ = superoxide; %DFI = DNA fragmentation index.

## 4 Discussion

The purpose of this study was to compare two methods of bull epididymal sperm collection, and the effect of cryopreservation on sperm quality. The results for sperm viability were similar for both cutting, and flushing methods and for both Right and Left sides, for either fresh or thawed sperm, apart from the proportion of dying sperm, which was greater in the cutting method. In contrast, in a previous study a difference in sperm viability was detected between testes from the same bulls (Goovaerts et al., 2006). Our results are in agreement with those of Kang et al. (2018), who reported that post-thaw sperm viability was not affected by flushing or mincing extraction methods. Fresh epididymal spermatozoa had a viability of 41.25 % when stored at 18–20 °C for 30 h (Bertol et al., 2013), while viability for fresh spermatozoa in our study was 35.1 % for method A and 37.6 % for method B. Both of these studies had a considerably lower viability than in the study by Turri et al. (2012) where viability was 77.2 % for the float-up method and 84.5 % for the flushing method. Similarly, in another study by Goovaerts et al. (2006), fresh spermatozoa were collected by making multiple incisions in the epididymis to allow the spermatozoa to exit. The resulting samples had total motility of 48.7 %, progressive motility 34.4 %, and live spermatozoa 85.35 %.

Total and progressive motility for fresh epididymal sperm samples in our study for method A were similar to the values reported by Cunha et al. (2019). However, our results for method B were higher. In contrast, Kang et al. (2018) had higher values for total motility, 89.5 %, and 91.4 % for flushing and mincing methods, respectively. Their results indicated higher total motility than membrane integrity (Kang et al., 2018, 2021; Cunha et al., 2019), which was also seen in the present study. A possible explanation for this observation is that spermatozoa with damaged membranes, i.e. membranes that become permeable to PI, may continue to be motile for a short period until the damage becomes incompatible with cell survival. A more accurate association might be seen by including SYBR14+/PI + in the correlation with motility, but this was not done in the present study.

Although ROS are known to be essential for sperm fertility, they can still inflict considerable damage on spermatozoa during storage, rendering them non-functional (Gibb et al., 2020). They can damage sperm membranes through lipid peroxidation and protein modification, as well as disrupting the electron transport chain and sperm mitochondria, resulting in a loss of sperm function and hence fertility (Gibb et al., 2020). Some potential damaging effects on membranes were seen in the present study, since SO production was negatively associated with membrane integrity, although curiously H_2_O_2_ production was not linked to a decrease in membrane integrity. Other studies reported an increase in ROS production during cryopreservation of bull semen due to the dilution of antioxidants in seminal plasma by the addition of cryoextender (Vigolo et al., 2022). Since the present study used epididymal spermatozoa, there was no additional antioxidative effect from seminal plasma; these results indicate that the production of ROS and their effects on spermatozoa is a complex subject that is not completely understood.

In addition, ROS can damage sperm DNA, inducing strand breaks and release of bases (Bollwein & Bittner, 2018), and sperm retention in the epididymis was reported to result in increased DNA damage (Evenson, 2022). Increasing ROS production causes increasing oxidative stress, reducing sperm chromatin integrity and male fertility (Cojkic et al., 2023). A negative correlation between %DFI and sperm motility was reported previously (Aleksander et al., 2003; Moradian et al., 2019) but was not apparent in our results. Gṻrler et al. (2016) described an increase in %DFI production concomitant with increased H_2_O_2_ production during freezing and thawing of bull spermatozoa. In contrast, %DFI was not correlated with H_2_O_2_ production in the present study, either in fresh or thawed samples. The same extender (Andromed) was used in the study by Gṻrler et al. (2016) and our study, and both studies used a vapour freezing method. Differences between the results of the two studies could be attributable to other methodological differences, breed and age of bull, or other variables.

There was a slight but significant negative association between sperm MI and %DFI in the present study, implying that spermatozoa with intact membranes may have less DNA fragmentation that spermatozoa with damaged membranes. This finding is in line with a similar result in a study evaluating the sperm quality of young bulls. There, DNA fragmentation was found to decrease and MI increased as the age of the bulls increased (Lima Verde et al., 2022). However, as indicated by Da Costa et al. (2021), the relevance of an association between MI and %DFI for fertility is limited. Spermatozoa with damaged membranes are unlikely to reach the oocyte and achieve fertilization. Therefore, it is actually the extent of DNA fragmentation in living sperm that is more relevant to the functionality of the sample. It was not possible to perform simultaneous measurement of membrane integrity and DNA fragmentation in the present study, but future studies should seek to incorporate this modification.

In a study comparing flushing or mincing to extract epididymal spermatozoa (Kang et al., 2021), the viability after thawing was 52.3 %, which is considerably higher than our post-thaw viability results (14.4 % for method A and 16.1 % for method B). Furthermore, Chaveiro et al. (2015) reported 78.1 % total motility, and 86.5 % viability in fresh sperm samples, and values of 56.9 %, and 64.5 %, respectively, after thawing. Cryopreservation protocols were different between the studies; Kang et al. (2021) used a sperm concentration of 40 × 10^6^ /mL, cooled the samples for 4 h, and froze them 3 cm above the surface of liquid nitrogen for 14 min, whereas Chaveiro et al. (2015) used a sperm concentration of 40 × 10^6^ /mL, cooled the samples at 5 °C for 2 h followed by cooling in a controlled freezer at -4 °C /min to -10 °C, and from -10 °C to -145 °C at a rate of – 40 °C /min. The results from these different studies suggest that the cryopreservation protocol used in the present study was not optimal; better post-thaw results might be obtained by modifying the vapour freezing method.

Collection of epididymal sperm could be beneficial for conservation breeding, as previously mentioned, when conventional semen collection methods are not possible. Therefore, it is important to develop protocols for retrieving and using the sperm samples (Leibo & Songsasen, 2002). In addition, there are other instances when such sperm samples could be useful, for example, when utilising material from freshly dead animals (Prieto et al., 2014), or if a male is unable to achieve an erection (Kapoor et al., 2015). Collecting the epididymal sperm enables the gametes from these animals to be used in insemination or for in vitro fertilisation (Chaveiro et al., 2015). However, the length of time that the sperm remain in the epididymis after death or removal of the testis affects sperm motility; sperm motility was low when spermatozoa were extracted 30 h after removal of the organs (Bertol et al., 2013). Furthermore, the presence of extraneous material, such as epithelial cells or blood, can be detrimental to sperm survival (Muñoz-Fuentes et al., 2014). Therefore, a method of sperm extraction that involves the least manipulation of the tissue would be preferred.

In conclusion, fresh epididymal spermatozoa collected either by incision or flushing the tail of epididymis are suitable for assisted reproduction technologies. However, it appears that more work is needed to optimise the cryopreservation protocol if it is intended to freeze such samples. Both extraction techniques were relatively simple to perform, without the need for sophisticated equipment, and could enable sperm samples to be extracted in the field. The incision method was less time-consuming than the flushing method, but in most cases yielded fewer spermatozoa than the flushing method, which could be important. Furthermore, the flushing method produced samples containing fewer dying spermatozoa.

## Funding

Ziyad Al-Kass was sponsored by the College of Veterinary Medicine - University of Mosul, Iraq. SE, TN and JMM were supported by the Swedish University of Agricultural Sciences.

## Data availability

All data are presented in the manuscript but will be supplied on reasonable request.

## Ethical statement

All co-authors contributed to this study, have read the final version, agree to its publication and accept responsibility for the work. All data are reported in the paper.

All procedures were performed in compliance with relevant laws and institutional guidelines. The appropriate institutional committee does not require to approve the use of slaughterhouse material, which is regarded as waste (please see attached document from the relevant committee).

## CRediT authorship contribution statement

**Ziyad Al-Kass:** Writing – original draft, Visualization, Investigation, Data curation, Conceptualization. **Sanna Eriksson:** Writing – review & editing, Investigation. **Jaana Peippo:** Writing – review & editing, Methodology. **Theodoros Ntallaris:** Writing – review & editing, Formal analysis. **Jane M. Morrell:** Writing – review & editing, Supervision, Resources, Methodology, Funding acquisition, Conceptualization.

## Declaration of competing interest

The authors declare the following financial interests/personal relationships which may be considered as potential competing interests: Jane Morrell reports financial support was provided by Seydlitz Foundation, Stockholm. If there are other authors, they declare that they have no known competing financial interests or personal relationships that could have appeared to influence the work reported in this paper.
